# The effect of home-based low-volume, high-intensity interval training on cardiorespiratory fitness, body composition and cardiometabolic health in women of normal body mass and those with overweight or obesity: protocol for a randomized controlled trial

**DOI:** 10.1186/s13102-019-0152-6

**Published:** 2019-12-30

**Authors:** Emmanuel Frimpong, Chloe Dafkin, Janine Donaldson, Aletta Maria Esterhuyse Millen, Rebecca Mary Meiring

**Affiliations:** 1 Movement Physiology Research Laboratory, School of Physiology, Faculty of Health Sciences, University of the Witwatersrand, Johannesburg, South Africa; 2 Endocrinology and Metabolism Research Laboratory, School of Physiology, Faculty of Health Sciences, University of the Witwatersrand, Johannesburg, South Africa; 3 Cardiovascular Pathophysiology and Genomics Research Unit, School of Physiology, Faculty of Health Sciences, University of the Witwatersrand, Johannesburg, South Africa; 4 Department of Exercise Sciences, Faculty of Science, The University of Auckland, Building 907-228, Suiter Street, Newmarket, Auckland, 1023 New Zealand

**Keywords:** High intensity low-volume interval training, Overweight, Obesity, Body composition, Cardiometabolic health, Physical activity, Exercise, Women

## Abstract

**Background:**

There is a high prevalence of women in South Africa with overweight and obesity which is associated with an increased risk of cardiometabolic disorders. Perceived barriers such as lack of time and motivation reduce engagement in beneficial activity behaviours for health. High-intensity interval training (HIIT) is a time-efficient and effective way to improve cardiometabolic risk profile regardless of a loss in body mass or change in body composition. This randomized controlled trial aims to determine the effects on cardiorespiratory fitness, body composition and cardiometabolic health and feasibility of a home-based 14-week HIIT program in women with overweight/obesity or normal body mass.

**Methods:**

One hundred and twenty women (18–40 years old) with a body mass index between 20 and 35 kg/m^2^, will be stratified according to their BMI (normal, BMI 20–24.9 kg/m^2^; or high BMI ≥25 kg/m^2^) and randomized into a HIIT exercising group (HIIT) or a non-exercising control group (CON). HIIT participants will perform exercises for 11 min/session six times per week for a period of 14 weeks. The 2 × 4 HIIT protocol will require a work phase of own-body weight exercise lasting 2 minutes (85% VO_2_peak), repeated four times and separated by a one-minute active rest phase (65% VO_2_peak). CON participants will be asked to maintain their normal habitual lifestyle. Outcomes of cardiorespiratory fitness, body composition, echocardiography, central blood pressure, arterial stiffness and biomarkers of cardiometabolic health will be measured before and after the 14-week intervention. Every 4 weeks during the intervention, an objective estimation of compliance to the study protocol will be assessed by measuring participant physical activity over 7 days using an Actigraph GT3X accelerometer.

**Discussion:**

Supervised laboratory-based HIIT interventions are effective in improving cardiometabolic health. More pragmatic exercise protocols may however show to be successful for mitigating barriers to the engagement in physical activity and exercise resulting in positive benefits to health. Investigation into home-based HIIT regimens are important in women, where globally the rising trend of overweight and obesity overshadows that of men. The results from this study may therefore inform future research on effective exercise prescription for women’s health.

**Trial registration:**

Pan African Clinical Trial Registry (www.pactr.org - id no: PACTR201806003434299), 6th June 2018.

## Background

Non-communicable diseases (NCDs), including overweight and obesity, are estimated to cause 71% of deaths worldwide and 51% of deaths in South Africa [1], a statistic which outnumbers those deaths caused by communicable diseases [2]. The most recent data from the South African National Health and Nutrition Examination Survey (SANHANES) reports that 64% of South African women older than 15 years, are overweight or obese (body mass index (BMI) > 25 kg/m^2^) and therefore at a greater risk of suffering from other NCDs [3]. These data are in line with global trends of obesity whereby more women than men are overweight and obese [4]. Excess weight gain or obesity is associated with concurrent increases in the incidence of hypertension, risk of type 2 diabetes, serum low density lipoprotein (LDL) cholesterol concentrations, and cardiovascular and metabolic disease risk [5, 6]. Obesity is also associated with several diseases in women including certain cancers (including breast and colon) [7–9], depression and sleep disturbances [10, 11], as well as all-cause mortality [12–14]. Hence it is not surprising that lifestyle interventions aimed at weight loss are still considered a cornerstone in the prevention and management of disease risk in women [15]. Following the World Health Assembly’s release of a comprehensive global monitoring framework and targets for prevention and control of NCDs [16], the South African National Department of Health (NDoH) also issued a five-year strategic plan for the prevention and control of NCDs between the years 2013–2017. The strategic plan aimed to achieve a 10% reduction in overweight and obesity, 20% reduction in elevated blood pressure and 10% increase in participation in physical activity (PA) [17] through lifestyle modifications by the year 2020 [18]. Public health strategies in South Africa have also emphasised increasing energy expenditure through an increase in daily PA and/or participation in exercise for maintaining ideal body mass. However, more research on engagement in and the effect of different possible exercise strategies on health outcomes is needed.

### Engagement in exercise and PA and barriers to participation in PA

Increasing participation in exercise and PA is associated with a decrease in body fat [19–22], which reduces one’s risk of obesity-related diseases [23, 24] and all-cause mortality [12, 14]. Current PA guidelines recommend that adults take part in at least 150 min of moderate intensity exercise or 75 min of vigorous intensity exercise per week for weight management [25]. However, only about 50% of healthy adults worldwide meet these recommended PA guidelines [3, 26]. Physical inactivity, which is defined as not meeting the recommended guidelines, is associated with hypertension, diabetes and obesity [27]. Lack of time [28], family responsibilities [29], fatigue or feelings of weakness, lack of motivation, confidence, self-discipline and unaffordable exercise programmes as well as not making exercise a priority and health issues are well-documented barriers to sufficient PA participation in healthy populations [29–32]. A survey of women aged 18–49 years, from low-income households in Latin America who were at risk of type 2 diabetes indicated that lack of willpower and energy were the most frequently perceived barriers to physical activity engagement [33]. In people with diagnosed obesity-related chronic diseases e.g. cardiovascular disease and type 2 diabetes, and who are referred to exercise-based rehabilitation programmes, independent engagement in exercise and physical activity behaviours remains low during the maintenance phases of rehabilitation [34–36]. Another well-established reason for poor engagement in long-term exercise maintenance programmes is due to lack of transport or the associated costs of transport to rehabilitation centres especially in lower income households [35]. Finally, women who are overweight or obese report that social influence and fear of injury are important barriers to exercise [33]. More research is therefore warranted to investigate pragmatic, convenient and time-saving physical activity and exercise interventions that will maximise overall health benefits, and that are feasible in engaging both healthy and clinical populations.

### High intensity interval training

High intensity interval training (HIIT), has received much recent attention in health literature as a time-saving, effective exercise modality for cardiometabolic health [37]. HIIT is characterised by bouts of vigorous activity separated by short rest intervals. These vigorous intensity exercise bouts typically require maintaining intensity thresholds of at least 85% of an individual’s maximum heart rate (HR), followed by active or passive rest at 65% maximum HR [37]. Although there are a myriad of protocols used, most evidence suggests that HIIT is equally if not more beneficial for cardiometabolic health compared to moderate intensity continuous training (MICT) [38]. Therefore, HIIT may offer an effective solution to people who struggle to maintain adequate levels of physical activity. The most commonly observed primary outcome measure in HIIT studies is a change in peak oxygen consumption (VO_2_peak) as an indirect measure of improvement in cardiovascular disease risk [39]. Various other improvements in cardiometabolic health markers are also observed in trials investigating HIIT in adults with overweight/obesity, including reductions in systolic and diastolic blood pressure [40, 41], reversal of dyslipidemia [42, 43], improvements in insulin resistance, glycated haemoglobin (HbA1c) and fasting blood glucose concentrations [43–45]. The improvements in cardiometabolic risk factors are observed following both short term (< 12 weeks) and long term (> 12 weeks) participation in HIIT interventions despite no significant change in body composition [40].

### Feasibility of HIIT interventions

To date, the vast majority of studies investigating the efficacy of HIIT in adults with overweight or obesity have used supervised, lab-based running and cycling protocols [40, 46, 47]. However, HIIT may be performed through various movements and is not limited to running or cycling. Other own-body weight exercises for use in HIIT have gained popularity and are easily conducted in the comfort of one’s home. Whether home-based HIIT activity produces similar cardiometabolic benefits to the classic running and cycling protocols needs investigation because understanding the feasibility of engagement in physical activity outside of research and clinic settings is important for understanding how to sustain health benefits. To our knowledge, only one recent study examined the effects of an unsupervised, home-based HIIT programme on body mass over 12 months in overweight adults [48]. Although overall long-term adherence to the intervention was significantly low (23% of participants fully adherent), those participants who adhered to the programme showed greater reductions in body mass compared to non-adherent individuals [48], with adherent HIIT individuals performing the exercises for between 21 and 24 min per week. It is currently unknown whether a home-based HIIT can produce improvements in risk factors for cardiovascular and metabolic diseases.

The difficulty in prescribing an adequate dose of HIIT for loss of fat mass is largely due to the variability in protocols used in interventions [49, 50]. Cowan et al. [51] have shown in women with abdominal obesity that continuous, aerobic exercise for 24 weeks resulted in a reduction in total and abdominal adipose tissue independent of the amount or intensity of exercise. Women in a low-intensity, low-volume group who exercised for 30 min per session showed similar reductions in adipose tissue compared to the high-volume, high-intensity group (40 min/session) and the high-volume, low-intensity (almost 60 min/session) group [51]. Some HIIT studies exceed the recommended guidelines for weekly vigorous physical activity, an amount of time which appears to be difficult to achieve in many populations [3, 26]. If sustained participation in any exercise is a primary factor in determining benefits to cardiovascular health, more studies of the feasibility of participation in low volume HIIT are needed. In addition, more randomized controlled trials using a healthy group comparator should be done to determine whether there are differences in the effects of HIIT between people with chronic disorders affecting cardiometabolic health and people free from chronic disorders [40]. These studies may guide further investigations into understanding the physiological mechanisms underlying the benefits previously seen in HIIT trials.

Globally, public health interventions to decrease the prevalence of high body mass are failing to yield the desired outcomes in individual countries [22, 52]. Alternative and feasible exercise interventions that lower the risk of developing NCDs are important to investigate in women, who may often face multiple barriers to regular exercise participation. This paper describes the protocol for a randomised controlled trial to primarily assess effects of a 14-week high-intensity low-volume interval training intervention on the cardiorespiratory fitness of women with overweight/obesity and women of normal body mass. The study secondly aims to determine the effect of the intervention on body composition and cardiometabolic health as well as the feasibility of the intervention in the groups of women.

## Methods/design

### Study objectives

This randomised controlled trial will compare the effects of a 14-week low-volume high-intensity interval training intervention on cardiorespiratory fitness, body composition and cardiometabolic health in women of normal body mass and women with overweight/obesity. In addition, adherence to and drop-out rate of the intervention will be measured to determine feasibility. We hypothesise that at the end of the 14-week intervention, women with overweight/obesity performing HIIT will have significant changes in their cardiorespiratory fitness and cardiometabolic health regardless of a change in body composition. We also expect that a low-volume HIIT intervention will be feasible in this cohort of women.

### Study design

**Fig. 1:**
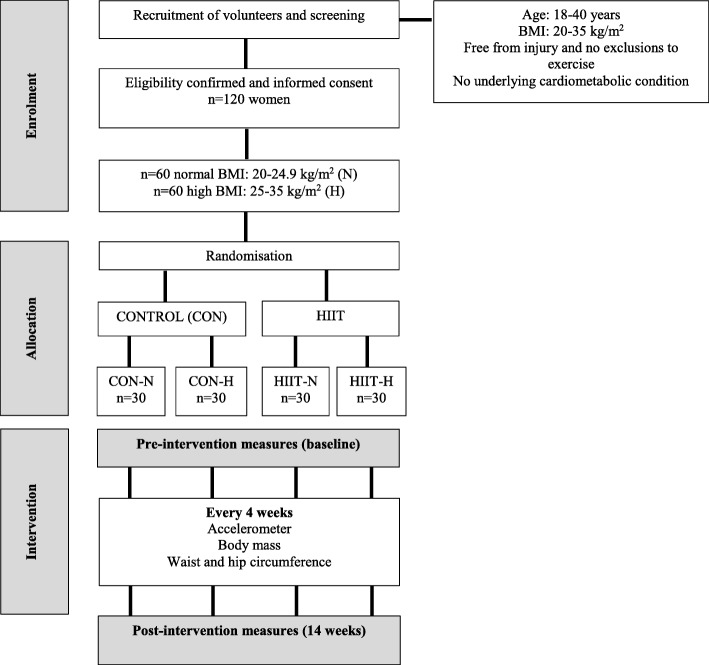
Flow of participants through the study

**Fig. 2:**
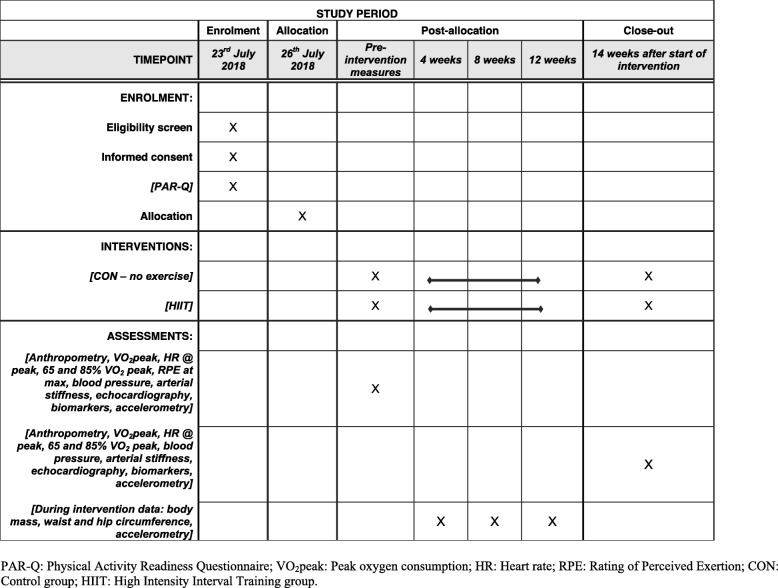
SPIRIT schedule of enrolment, intervention and assessments for the duration of the study

This study will be a randomized controlled trial (Fig. 1), where equal numbers of participants (normal and high BMI) will be assigned to either a HIIT group (HIIT) or a control (CON) group. The study has been designed using the SPIRIT guidelines (Fig. 2 and Additional file 1). The reporting of the trial will be guided by the CONSORT statement for clinical trials. Women in the HIIT group will complete a home-based high intensity exercise program for 14 weeks. The control group will be asked to maintain their current activity and dietary behaviours. Physiological measurements of cardiorespiratory fitness, body composition and cardiometabolic health will be done at baseline and on completion of the intervention at the 14th week. Habitual activity behaviours will be determined objectively using accelerometers for a week prior to the start of the intervention and for a week following the intervention. During the intervention, monitoring of and adherence to the HIIT protocol will also be assessed with accelerometers and an exercise diary. The study has been approved by the University of the Witwatersrand Human Research Ethics Committee (protocol number: M180253). Informed consent will be obtained from all participants prior to entry into the study. The trial has been registered on the Pan African Clinical Trial Registry (www.pactr.org - id no: PACTR201806003434299).

### Study site

The study measures will be conducted at the Movement Physiology Research Laboratory in the School of Physiology in Johannesburg, South Africa. The laboratory is situated in Parktown in the Faculty of Health Sciences, University of the Witwatersrand. Participants will conduct the 14-week intervention outside of the laboratory setting.

### Recruitment of participants

Volunteers will be recruited from the academic community of the University as well as from the surrounding central business district. Study advertising will be placed on the online University newsletter, social media platforms such as Facebook, through direct invitation by the research team via their teaching roles and posters with study information will be displayed throughout the University’s faculties. The expected time frame for recruitment of participants is anticipated to take 18–24 months. Eligible volunteers will be enrolled into the study as and when they volunteer. Participants will be compensated for their costs incurred travelling to the laboratory for assessments. Recruitment of participants started on 1st July 2018 and is ongoing, and data collection is in progress.

### Study participants/eligibility criteria

Women between the ages of 18 and 40 years will be invited to participate in the study. Women will be included if they have a BMI between 20 and 35 kg/m^2^ (normal to obese BMI categories) and are free from injury and/or known clinical conditions which would exclude them from partaking in exercise as assessed using the physical activity readiness questionnaire (PAR-Q). Known clinical conditions include family history of sudden death, uncontrolled hypertension, type I or type II diabetes and cardiovascular disease. No pre-requisite training background is required however participants will be excluded from the study if they report engaging in competitive exercise or take part in a structured exercise programme that has been prescribed by a physical trainer or other health-related exercise professional. Exclusion criteria are also women with a BMI < 20 kg/m^2^ or > 35 kg/m^2^, and if they report any diagnosed underlying cardiometabolic condition including hypertension, diabetes or cardiovascular disease. All volunteers will be informed verbally and in writing of the procedures of the study before signing an informed consent form and completing screening questionnaires. Participants will be asked to maintain their habitual diet throughout the study.

#### Sample size determination

Based on a small effect size of 0.20 (Cohen’s f) for an improvement in relative VO_2_peak after HIIT versus no HIIT [42], performing a priori sample size calculation for a repeated measures analysis of variance with an expected correlation of 0.50 between measurements, alpha of 0.05 and 85% power of detection, a total sample size of 60 women will be required (G*Power, version 3.1.9.2). The sample size will be doubled to account for further planned analysis of secondary outcomes in sub-groups of normal and high BMI women (HIIT normal BMI, HIIT high BMI, CON normal BMI, CON high BMI). The target sample size will be therefore be 120 women exclusive of drop outs.

### Outcome measures

The primary outcome of the trial is a change in cardiorespiratory fitness after the 14-week HIIT intervention. Secondary outcomes are changes in body composition, cardiovascular health and metabolic biomarkers of health, as well as measures of feasibility and physical activity and sedentary behaviour levels. Both primary and secondary outcomes will be measured before and after the 14-week intervention (Fig. 2).

Table 1 summarises the outcome measures to be made and the time points at which they will be measured during the study.

**Table 1:** Indication of physiological variables measured and timepoints of each measurement

Variable	Timepoints measured
*Primary outcome*	
Peak VO_2_, HR at peak VO_2_, HR at 65 and 85% peak VO_2_	Baseline, post-intervention
*Secondary outcomes*	
Anthropometry	
Skinfolds	Baseline, post-intervention
Body mass and waist:hip	Baseline, every 4 weeks during intervention, post-intervention
Brachial blood pressure	Baseline, post-intervention
Central blood pressure and arterial stiffness	Baseline, post-intervention
Echocardiography	Baseline, post-intervention
Biomarkers of metabolic and cardiovascular health	Baseline, post-intervention
Feasibility (adherence and dropout rate)	Baseline, every 4 weeks during intervention, post-intervention
Accelerometry	Baseline, every 4 weeks during intervention, post-intervention

*VO*_*2*_*peak* Peak oxygen consumption, *HR* Heart rate

### Study procedures

#### Study visits

Volunteers will be required to visit the laboratory for an information session on the study. Eligible participants will then visit the laboratory to have their baseline and post-intervention measures taken. Participants in the exercising group will perform the HIIT program for 14 weeks in their own homes. During the intervention all participants will also visit the laboratory every 4 weeks (4th, 8th, and 12th weeks) to have an accelerometer fitted for measures of seven-day habitual activity. At these visits, body mass and waist-hip ratio will also be measured. Participants will be asked to refrain from consuming alcohol or caffeine for at least 24 h and food for at least 8 hours (eight-hour fast) before visiting the laboratory. Participants will also be asked to refrain from performing strenuous exercise 48 h prior to their measurement days. Post-intervention measurements will be performed a minimum of 48 h and a maximum of 4 days after the end of the HIIT intervention.

#### Randomisation

All eligible women will be classified as being in either one of two BMI groups (normal BMI: 20-24.9 kg/m^2^, *n* = 60; high BMI: 25-35 kg/m^2^, *n* = 60). Women will then be randomized into either a control group (CON) or a group that will follow the HIIT protocol (HIIT) resulting in four groups of women: high BMI control (CON-H; *n* = 30), normal BMI control (CON-N; n = 30), high BMI HIIT (HIIT-H; n = 30) and normal BMI HIIT (HIIT-N; n = 30). The randomisation procedure for the intervention groups will be done using Microsoft Excel RANDBETWEEN function. The RANDBETWEEN function (=RANDBETWEEN (1,2)) is able to assign participants into one of the two groups, in this instance 1 = CON and 2 = HIIT. Two random sequences of 120 numbers each (group 1 or 2) is generated for each BMI category (high and normal). The sequences were generated by an investigator who is not involved in the data collection procedures. Upon enrolment of a participant into the study, the group allocation of the participant is revealed to a non-blinded research assistant responsible for data collection.

#### Blinding

The researcher collecting data will not be blinded to the group in which each participant is allocated because they will take all pre-, during and post-intervention measures from the participants and will monitor adherence to the HIIT intervention for the duration of the 14 weeks. An investigator and/or statistician will analyse data that has had the group allocation labels removed.

### Maximal exertion test and determination of the intensity of the home-based HIIT exercises

All participants will undergo a maximal exertion test for the determination of measures of cardiorespiratory fitness and to determine the relative intensity (heart rate and rating of perceived exertion) at which the HIIT intervention should be performed. The maximal exercise test will take place at the pre-intervention visit and at the end of the 14-week intervention. A progressive incremental exercise test to volitional fatigue on a motorised treadmill will be performed to determine maximal aerobic capacity (relative VO_2_peak). An exercise physiologist will monitor the test and test variables. The participants will first perform a five-minute warm up (0% gradient and self-selected speed). A Balke-Ware treadmill protocol will be used [53]. During the test the treadmill speed will stay constant at 5.3 km/h while the incline will increase by 1% every minute until the participant reaches exhaustion or the test is terminated. Participants will be required to wear a face mask (soft silicone for comfortable fit) during the test for the collection of expired respiratory gases. Breath-by-breath oxygen consumption and energy expenditure will be determined using a computerized analyzer (Quark ergo, COSMED, Rome, Italy). Heart rate will also be measured throughout the test using a wireless heart rate monitor connected to the same computerized analyzer. Rating of perceived exertion (RPE) will be monitored every 2 minutes during the test using the 6–20 Borg scale [54, 55]. The test will be terminated if the participant reaches volitional fatigue and/or if any of the test termination criteria appear as outlined in the American College of Sports Medicine (ACSM) test termination criteria [56]. The test will be considered a maximal effort if (i) the measured oxygen consumption does not increase by more than 150 ml per successive workload, (ii) a respiratory quotient (R) value equal to or above 1.15 is reached, (iii) heart rate is more than 90% of the age-predicted maximal heart rate and (iv) the RPE is above 19 on the 6–20 Borg scale. From the maximal exercise test, peak uptake of oxygen indexed to body mass (relative VO_2_peak in ml/min.kg^− 1^) will be obtained and heart rate and estimated RPE at 85% VO_2_peak_*,*_ and 65% VO_2_peak will be calculated to determine the intensities of the HIIT work phase and the HIIT active rest phase respectively.

### The home-based HIIT intervention

The rating of perceived exertion and heart rate that occurred at 65 and 85% VO_2_peak during the maximal exercise test will be used as the targets for the intensity of the HIIT home-based intervention. The HIIT intervention will be thoroughly explained to each participant at an initial laboratory visit to ensure they understand the required effort needed to perform the HIIT sessions. Participants will receive an annotated diagrammatic sheet that explains what the exercises look like and how the exercises should be performed (Additional file 1) and they will also be provided with online resources for further guidance.

The protocol implemented will be a 2 × 4 HIIT (Table 2). A 2 × 4 HIIT requires a work phase lasting 2 minutes each which is repeated four times. During work phases participants are required to maintain a heart rate of 85% of their respective relative VO_2_peak. Between work phases (each 2 minutes of HIIT), a one-minute active rest phase is performed by maintaining movement i.e. by walking in the area/stepping on the spot at an RPE/HR corresponding to 65% of their relative VO_2_peak as determined from the maximal exercise test. For variation, four types of HIIT exercises, lasting 30s each, will make up each two-minute HIIT work phase. A total of eight types of own-body weight exercise will be performed. The exercises will be a combination of exercises such as high-knees, alternating backward lunges, squat jumps, inch-worms, sumo squats, running step-ups, mountain climbers and burpees. Considering a 2 × 4 required completion of exercise including a one-minute active rest phase, a total exercise time of 11 min per session will be completed. Participants in the HIIT group will be asked to perform a HIIT session six times per week on any days that suit them best (total of 66 min per week) for 14 weeks i.e. once a day on 6 days of the week or twice per day on 3 days of the week.

**Table 2:** Example of the timeline and intensity of each of the phases in the home-based HIIT protocol

	Phase							
	Exercise 1	Rest 1	Exercise 2	Rest 2	Exercise 3	Rest 3	Exercise 4	Rest 4
Intensity (% VO_2_peak)	85	65	85	65	85	65	85	65
Time (min)	0–2:00	2:00–3:30	3:30–5:30	5:30–7:00	7:00–9:00	9:00–10:30	10:30–12:30	12:30–14:00
	Running Step-ups	Walk/stepping	Squat jump	Walk/stepping	Running Step-ups	Walk/stepping	Squat jump	Walk/stepping
	Sumo squat		Inch worm		Sumo squat		Inch worm	
	Mountain climbers		Alternating lunges		Mountain climbers		Alternating lunges	
	Burpees		High knees		Burpees		High knees	

*VO*_*2*_*peak* Peak oxygen consumption

### Control group

The CON participants will undergo the maximal exertion treadmill test but will not perform the HIIT exercises over the 14-weeks. Instead they will be asked to maintain their habitual activity and dietary habits at the time of recruitment and to not start any new exercise regimen during the 14 weeks. However, at the end of the 14-week follow up, participants will have the chance to receive the list of the HIIT exercises including their training intensities (from the maximal exertion test) and training frequencies to perform at their leisure. The research assistant will demonstrate the exercises to them.

### Monitoring of adherence to the home-based HIIT protocol

During the intervention, all participants will be asked to wear an Actigraph wGT3X-BT accelerometer (Actigraph, LLC, Fort Walton Beach, FL, USA) for 7 days every 4 weeks as an objective measure of compliance to the intervention. The accelerometer data will be used to determine whether the total amount of time spent in vigorous intensity activity is different between the HIIT and CON. In addition, participants will be provided with an exercise diary and asked to log their exercise sessions as well as report on the rating of perceived exertion (a chart will be provided in the diary) for each session. Weekly mobile text messages will be sent to all participants including those in the control group, reminding them to maintain their compliance to the intervention. The Actigraph is a tri-axial accelerometer worn on the hip attached to an elasticated Velcro belt. The Actigraph will be provided to each participant for 1 week every 4 weeks during the intervention. Participants will be asked to wear the Actigraph around their waist for seven consecutive days of assessment for 24 h a day during the week of activity monitoring. Participants will be asked to only remove the Actigraph during showering, bathing or swimming activity. The time and duration that the Actigraph is removed for any reason will also need to be noted by the participant in their exercise diary.

### Data collection

#### Cardiorespiratory fitness

Measures of peak and submaximal relative oxygen consumption and heart rate will be determined as measures of cardiorespiratory fitness. These data will be collected using the maximal treadmill test described earlier, before the start of and at the end of the 14-week intervention.

#### Feasibility

Completion, dropout rates and intervention adherence will be used to determine the feasibility of the low-volume home-based HIIT intervention [57]. Feasibility will be reported using the following 1) the number of drop-outs after the intervention as a proportion of the number of recruited participants, 2) the number of HIIT sessions completed over the 14-week intervention period and 3) the number of participants who report not achieving the desired exertion level during the HIIT sessions at home. Data for points 2 and 3 will be collected using the exercise diaries.

#### Anthropometry/body composition

Various measures of body composition will be taken. Stature and mass will be assessed to the nearest 0.1 cm and 0.1 kg respectively and used to calculate body mass index (BMI). Waist and hip circumference will be measured to the nearest 0.1 cm using standard approaches. Body fat percentage will be calculated using the sum of four skinfolds (triceps, biceps, suprailliac and subscapular) [58]. Skin-fold thickness will be measured to the nearest 0.1 mm using Harpenden callipers (Baty International, West Sussex, UK). The same researcher will perform the measurement of skinfold thickness and the measurements will be taken after an overnight fast. Measurements will be taken twice and an average of the two values recorded unless the second measure is not within 5% of the first skinfold measure, then a third measure will be taken, with the median value then being recorded. Skinfold sites will also be measured in succession. Skinfold measurements will not be taken if the participant reports having recently exercised, been in the sauna, been swimming or has showered (less than 2 h since).

#### Cardiovascular health measures

##### Brachial blood pressure

Resting brachial blood pressure (BP) will be measured prior to any testing or exercise after a five-minute rest period. The average of two measurements will be taken with an automated BP monitor (SpaceLabs, Redmond, WA). If the first two measures for either the systolic or diastolic blood pressures differ by more than 5 mmHg, a third measurement will be taken to determine the mean blood pressure.

##### Central blood pressure and arterial stiffness

Pulse wave velocity (PWV), aortic reflected wave index, central aortic BP and its determinants (reflected and forward wave pressures) will be determined using commercially available hardware and software. After resting for 15 min in the supine position, the radial waveform will be recorded by applanation tonometry. A high-fidelity SPC-301 micromanometer (Millar Instrument, Inc., Houston, Texas), interfaced with a computer utilizing SphygmoCor software, version 9.0 (AtCor Medical Pty. Ltd., West Ryde, New South Wales, Australia) will be employed. The waveform will be calibrated by auscultatory measurement of the brachial BP. From the radial pressure waveform signal the SphygmoCor software calculates the aortic pressure waveform by means of a validated generalized transfer function. Aortic pulse pressure and central to brachial pulse pressure amplification ratio (PPamp) will be calculated. The magnitude of the forward and reflected wave components of the aortic pressure waveform will be determined by wave separation analysis using a modified triangular waveform (SphymoCor software). Aortic PWV will be measured by sequential recordings of the arterial pressure waveform at the carotid and the femoral arteries. Aortic PWV will be calculated as the ratio of the distance in meters to the transit time in seconds.

##### Echocardiography

Echocardiographic measurements will be performed using an ultrasound (Acuson S2000, Siemens Healthcare GmbH, Germany) with the participant in the partial left decubitus position. Left ventricular (LV) dimensions will be determined using two-dimensional directed M-mode echocardiography in the short axis view and these recordings will be analysed according to the American Society of Echocardiography convention. M-mode images will be obtained perpendicular to the posterior wall and as close to the mitral leaflet as possible without images of the mitral leaflet appearing. The interventricular septal wall thickness (IVS), posterior wall thickness (PWT) and internal dimensions of the left ventricle will all be measured at both end diastole and end systole. Left ventricular diastolic function will be assessed using pulsed wave Doppler by assessing the mitral inflow at rest in the apical four chamber view. Pulse wave Doppler recordings of transmitral velocity will be obtained during early (E) and late (atrial-A) periods of left ventricular diastolic inflow, and expressed as the E/A ratio. Tissue Doppler imaging (TDI) will be used to measure the motion of the mitral valve annulus in the apical four-chamber view. The peak relaxation velocities during early (e’) and late (atrial) (a’) diastole will be obtained. Measures of left ventricular diastolic function will be expressed as the E/e’ ratio and the ratio of early to late mitral annular velocity (e’/a’). Cardiac systolic function will also be assessed by using two-dimensional speckle tracking derived myocardial deformation (strain and strain rate). Speckle tracking analysis will be performed on parasternal short axis images for circumferential strain and apical four chamber images for longitudinal strain. Peak strain and peak strain rate will be recorded.

#### Cardiometabolic biomarkers of health

After an overnight fast, 15 millilitres of blood will be obtained from the antecubital vein. Blood samples will be centrifuged, and plasma and serum will be stored at − 80 °C for later analysis. A blood sample via fingerprick will be also be analysed using a Point-Of-Care analyser (CardioCheck Plus, PTS diagnostics, Indianapolis, USA) for, total cholesterol, high-density lipoprotein (HDL) cholesterol, low-density lipoprotein (LDL) cholesterol and triglycerides (TGs). Using the stored plasma and serum samples, the concentrations of various inflammatory markers and markers of metabolic and cardiovascular health will be determined. ELISA tests (Quantikine® HS, R & D Systems, Inc., Minneapolis, MS, USA) will be used to determine the concentrations of glucose, insulin, interleukin-6 (IL-6), high sensitivity C-reactive protein (hs-CRP), as well as adipokines, including adiponectin, leptin, and chemerin. Endothelial dysfunction will be determined by means of measuring circulating adhesion molecules including ICAM-1, VCAM-1 and E-selectin also using commercial ELISA kits. Insulin sensitivity will be estimated using the homeostasis model assessment of insulin resistance (HOMA-IR) [59].

### Statistical analysis

Data will be analysed using Stata (version 15.1, StataCorp LLC, TX, USA). Data will be expressed as mean (SD) or as median (IQR). Data will be tested for normality (Shapiro-Wilks test) and relevant parametric or non-parametric tests will be applied to the testing of hypotheses. Patient characteristics will be reported for all those recruited into the study as well as those remaining in the study at the end of the intervention using descriptive statistics (mean and standard deviation or median and interquartile range or percentages). A linear mixed model analysis will be used to determine the effects of the HIIT intervention on body composition measures and cardiometabolic parameters with fixed factors of exercise group and time and a random factor of participant. The variance-covariance structures will be selected based on Bayes Information Criterion and the unstructured variance-covariance used. In the case of data that is not normally distributed a generalised linear mixed model will be used. Secondary outcome data between the four groups of women will be analysed in a similar way. Participant age and baseline physical activity level will be included in the models as covariates. Statistical significance will be set at 0.05 and effect sizes will also be reported.

## Discussion

This study intends to determine whether 66 min per week of own-body weight HIIT (which is 9 minutes/week less than the current WHO recommended guidelines of 75 min of vigorous activity per week) performed at home has the ability to 1) improve cardiorespiratory fitness, 2) result in cardiometabolic benefits regardless of BMI and 3) be a feasible method of engaging in exercise. The study also aims to determine whether the body composition and cardiometabolic changes in women with overweight/obesity and normal body mass are different compared to the non-exercising controls. There is evidence that short duration high intensity exercise has benefits to certain aspects of cardiovascular health [60, 61] however these studies were conducted in healthy males [60] and in patients with metabolic syndrome [61] respectively. Supervised HIIT has been shown to be as beneficial if not more beneficial compared to MICT on various parameters of health including blood lipid profiles, fasting glucose and insulin levels and blood pressure [37, 40, 41]. The time dedicated to supervised HIIT interventions however is often over and above the minimum recommended dose prescribed for the maintenance of cardiometabolic health [40]. As one of the reasons cited for people not participating in regular exercise being a lack of available time [28], investigation into the efficacy of low volume but high intensity exercise on cardiometabolic health is needed to determine whether it is possible for people to minimise their required engagement in exercise for health including the need for generally healthy people to be supervised. HIIT may be especially beneficial for women, a group that experiences inequity in physical activity engagement [62]. More long-term controlled trials investigating other markers of cardiometabolic health risk are needed in women to begin to explore the mechanisms for the efficacy of HIIT. Furthermore, most studies have used treadmill or cycling protocols and if a paradigm shift in the general population is to occur towards increasing levels of physical activity in habitual life, then more studies are needed on the efficacy of alternative exercise protocols on health in various populations in realistic settings. Only one study has investigated the effect of a one-year home-based HIIT on health in overweight adults. However the study participants showed poor compliance to the intervention and healthy controls were not included in the study [48].

We acknowledge that a potential limitation, as previously reported [48], will be compliance to the intervention. However, we aim to improve compliance by sending mobile text messages to the participants encouraging them to maintain engagement with the intervention. Motivational messaging is a strategy that has been used in other remote interventional trials [63–65] that have used behavioural therapy (motivation and counselling) which is complementary to exercise prescription. Another limitation to the study is that due to financial constraints it is not possible to provide a heart rate monitor to each participant for the monitoring of heart rate during the exercise sessions for the duration of the study. However, the first HIIT session will be conducted in the laboratory along with an exercise physiologist who will explain the protocol and indicate the individualised RPE at which the participants should exercise throughout the study. RPE using the Borg scale is a valid method for monitoring and prescribing exercise intensity regardless of sex, age, exercise modality or physical activity status [54].

The present study has the potential to inform future research on prescribing exercise therapy for people who are time-constrained. HIIT allows people to be flexible with engaging in physical activity instead of it becoming a burden to perform; and may encourage individuals who do not meet the physical activity guidelines to adopt a low-volume home-based HIIT programme for increasing engagement in physical activity levels for improved cardiometabolic and overall health. In addition, in comparing effects of HIIT between high and normal BMI groups, the current study will possibly identify future areas of mechanistic research as to how home-based own-body weight HIIT confers benefits to cardiometabolic health. The feasibility of low-volume, high-intensity exercise in overweight women could potentially mitigate some of the barriers to performing physical activity in a population that is at risk of cardiometabolic disease. Since HIIT is an effective exercise modality for the maintenance of cardiometabolic health, investigating the adherence to a home-based protocol is an important factor in understanding whether engagement in HIIT is in fact sustainable beyond the laboratory.

## Supplementary information

**Additional file 1.** SPIRIT 2013 Checklist: Recommended items to address in a clinical trial protocol and related documents*.

## Data Availability

Not applicable.

## Abbreviations

ACSM: American College of Sports Medicine
BMI: Body mass index
BP: Blood pressure
ECG: Electrocardiograph
HbA1c: Glycated haemoglobin
HDL: High density Lipoprotein
HIIT: High intensity interval training
HOMA-IR: Homeostasis model assessment of insulin resistance
HR: Heart rate
hs-CRP: High sensitivity C-reactive protein
IL-6: Interleukin-6
IVS: Interventricular septal wall thickness
LDL: Low density Lipoprotein
LV: Left ventricular
MICT: Moderate intensity continuous training
MVPA: Moderate-vigorous intensity physical activity
NCDs: Non-communicable diseases
NDoH: National Department of Health
PA: Physical activity
PPamp: Brachial pulse pressure amplification ratio
PWT: Posterior wall thickness
PWV: Pulse wave velocity
RPE: Rating of perceived exertion
SANHANES: South African National Health and Nutrition Examination Survey
TDI: Tissue Doppler imaging
TGs: Triglycerides
VO_2_peak: Peak oxygen consumption
WHO: World Health Organisation
